# Asymptomatic urinary tract infection among pregnant women attending the antenatal clinic of Hawassa Referral Hospital, Southern Ethiopia

**DOI:** 10.1186/1756-0500-7-155

**Published:** 2014-03-17

**Authors:** Endale Tadesse, Million Teshome, Yared Merid, Belayhun Kibret, Techalew Shimelis

**Affiliations:** 1 Department of Medical Laboratory Science, College of Medicine and Health Sciences, Hawassa University, P.O. Box 1560, Hawassa, Ethiopia; 2 Gynaecology and Obstetrics Unit, College of Medicine and Health Sciences, Hawassa University, P.O. Box 1560, Hawassa, Ethiopia; 3 Medical Microbiology and Parasitology Unit, College of Medicine and Health Sciences, Hawassa University, P.O. Box 1560, Hawassa, Ethiopia; 4 Biochemistry Unit, College of Medicine and Health Sciences, Hawassa University, P.O. Box 1560, Hawassa, Ethiopia

**Keywords:** Asymptomatic bacteriuria, Urinary tract infection, Pregnant women

## Abstract

**Background:**

Untreated asymptomatic bacteriuria (ASB) during pregnancy may cause serious complications including pyelonephritis and delivery of premature or low-birth-weight infants. However, little is known about asymptomatic bacteriuria in pregnancy in Ethiopia. This study aimed to assess the prevalence of asymptomatic bacteriuria, bacterial agents, and their antibiotic susceptibility pattern in pregnant women attending antenatal clinic of the Hawassa Teaching and Referral Hospital.

**Methods:**

A cross-sectional study was conducted in a total of 244 pregnant women with no sign and symptom of urinary tract infection from March 2012 to September 2012. Clean catch mid-stream urine samples were collected from all study participants using sterile containers. Urine samples were cultured using standard bacteriological methods. Identification of suspected colonies and antibiotic sensitivity testing were done.

**Result:**

Out of 244 pregnant women, 46(18.8%) were positive for asymptomatic bacteriuria (Colony Forming Unit ≥ 10^5^/mL). There was no difference in prevalence of asymptomatic bacteriuria with respect to age (p = 0.07) and trimester (p = 0.27).The most frequently isolated bacteria were coagulase negative *Staphylococcus* (32.6%), followed by *Escherichia coli* (26.1%), and *Staphylococcus auerus* (13%). The susceptibility rate of bacterial isolate was highest for norfloxacin (64.7%) and lowest for ampicillin (17.6%).

**Conclusion:**

The high prevalence of ASB in pregnant women warrant the need to screen all pregnant women and treat those infected with appropriate antimicrobial regimens in order to reduce its complications.

## Background

Urinary tract infection (UTI) is a common health problem among women compared with men due to shorter urethra, closer proximity of the anus with vagina, and pathogen entry facilitated by sexual activity [1,2]. It is estimated that one in three women of childbearing age contracts UTI [3], which may manifest symptoms or remain asymptomatic. Pregnant women are more susceptible to UTI, owing to altered anatomical and physiological state during pregnancy [2].

Asymptomatic bacteriuria (ASB) is a presence of a significant quantity of bacteria in a properly collected urine specimen from a person without symptoms or signs of UTI [4]. A prevalence of asymptomatic UTI ranging from 2% to 15% was reported compared to symptomatic UTI in pregnant women [5]. Socioeconomic factors and past history of asymptomatic urinary tract infection were related with UTI in pregnancy [6,7]. About 30% of women with untreated asymptomatic bacteriuria during pregnancy develop pyelonephritis [3], which might lead to delivery of premature or low-birth-weight infants [8,9].

In Ethiopia, studies reported prevalence of asymptomatic bacteria among pregnant women to range from 7% to 10.6% [10,11]. Bacteria including *Escherichia coli*, *Staphylococcus aureus*, *Providencia* species, *Klebsiella* species and *Staphylococcus saprophyticus*[12-14] were commonly isolated from pregnant women with UTI. However, in most developing countries including Ethiopia, screening for ASB in pregnancy is not considered as an essential part of antenatal care. Little is also known regarding the epidemiology of asymptomatic bacteriuria in pregnant women in southern Ethiopia. This study therefore aimed to determine the magnitude of asymptomatic bacteriuria, type of bacterial etiology and their antibiotic susceptibility pattern in pregnant women attending antenatal clinic of the Hawassa Teaching and Referral Hospital, southern Ethiopia.

## Methods

A cross-sectional study was conducted at Hawassa Teaching and Referral Hospital, southern Ethiopia from March 2012 to September 2012. The hospital is located at Hawassa, which is the capital city of South Nations, Nationalities and People’s Regional state, and the largest in the administrative region.

The study population comprised of all pregnant women attending the antenatal clinic in the hospital during the study period. However, pregnant women who were on antibiotic treatment two weeks prior to data collection and those with clinical signs and symptoms of UTI were excluded. The sample size was estimated using 6% prevalence [10], 3% margin of error and 95% confidence level. In total, 244 pregnant women were included in the study.

Demographic data including age and pregnancy gestational age was collected using questionnaires. Clean catch mid-stream urine samples were collected from all participants using wide-mouthed sterile capped container. The specimen was promptly transported to Microbiology laboratory and cultured within one hour of collection. Briefly, about 0.002 ml of samples were inoculated on Cystine Lysine Electrolyte Deficient (CLED) agar, Mannitol salt agar (MSA), and blood agar plate (Oxoid Basingstoke, UK) and incubated aerobically at 37°C for 24 hours. Bacterial growth was categorized and interpreted on the basis of colony forming units (CFU) as follows: equal to or greater than 10^5^ CFU/ml was considered as positive; less than 10^5^ CFU/ml were contamination. Bacterial isolates were identified based on their characteristic appearance, Gram reaction and pattern of biochemical reactions.

Antimicrobial susceptibility test was performed using disk-diffusion method on Müller-Hinton agar medium (Oxoid Basingstoke, UK) according to the instruction of the Clinical and Laboratory Standards Institute [15]. Antibiotic discs tested against bacterial isolates were trimethoprim-sulphamethoxazole (SXT, 25 μg), gentamicin (CN, 10 μg), cefotaxime (CTX, 30 μg), ampicillin (AMP, 10 μg), penicillin (P, 10 IU), vancomycin (VA, 30 μg) and norfloxacin (NOR, 10 μg) (Oxoid Basingstoke, UK). Reference strains of *E. coli* (ATCC- 25922) and *S. aureus* (ATCC- 25923) were tested as controls.

Data entry and analysis was performed using SPSS V-16. Descriptive summaries were presented, and Chi-square test (x^2^) was used to assess difference between proportions. P-value less than 0.05 was considered as statistically significant.

The study was ethically approved by the Institutional Review Board of College of Medicine and Health Sciences, Hawassa University. Participation was fully voluntary; and consent was obtained from all participants. Any information obtained during the study was kept confidential, and Doctors manage those women with UTI.

## Results

Single urine samples collected from a total of 244 pregnant women were examined for ASB. The mean age of the study participants was 26.13 (SD = 6.37, range = 15–49). The majority of the pregnant women were in their 3rd trimester (45.1%) and 2nd trimester (41%). Of the analyzed urine samples, 46 (18.8%) were positive for significant bacteriuria (CFU ≥ 10^5^/mL). The rate of isolation was higher in the age group ≥ 35 year (41.67%), though the difference was not statistically significant (p = 0.07). Women at their 2nd and 3rd trimester had a respective 20% and 20.9% rates of ASB, though this difference was also statistically non-significant (p = 0.27) (Table 1).

**Table 1:** Distribution of ASB by age group and trimester among pregnant women at Hawassa Teaching and Referral Hospital, 2012

**Characteristic**	**Total tested (%)**	**Number (%) positive**	**Chi-square value**	**p-value**
**Age (years)**				
15- 24	90 (36.9)	19(21.1)	5.43	0.07
25-34	142(58.2)	22(15.5)		
≥35	12(4.9)	5(41.67)		
**Total**	**244(100)**	**46(18.8)**		
**Trimester**				
1st trimester	34 (13.9)	3(8.8)	2.626	0.27
2nd trimester	100 (41.0)	20(20)		
3rd trimester	110 (45.1)	23(20.9)		

The majority (89.1%) of the infected women had single infection; while 10.9% were dually infected, which makes the total number of bacterial isolates 51 (Table 2). Of the bacterial isolates, 26 (51%) were Gram-positive bacteria and the rest 25 (49%) were Gram-negative bacteria. The predominant bacterial species were coagulase negative *Staphylococcus* (CoNs) (32.6%), *E. coli* (26.1%), *S. auerus* (13%), *Enterobacter* species 4(8.7), and *Klebsiella* species 3(6.5%). CoNS and *E. coli* was responsible for 4 (8.7%) of mixed infection.

**Table 2:** Pattern of single and mixed bacterial infections among pregnant women attending Hawassa Teaching and Referral Hospital, 2012

**Pattern of single and mixed bacterial isolates**	**Frequency (%)**
**Single infection**	
*Staphylococcus auerus*	6(13)
CoNS	15(32.6)
*Escherichia coli*	12(26.1)
*Klebsiella* species	3(6.5)
*Pseudomonas aeruginosa*	1(2.2)
*Enterobacter* species	4(8.7)
**Mixed infection**	
CoNS and *Escherichia coli*	4(8.7)
*Staphylococcus auerus* and *Citrobacter* species	1(2.2)
**Total**	**46 (100)**

CoNS = Coagulase negative Staphylococci.

Antimicrobial susceptibility of isolated bacteria showed that: norfloxacin (64.7%), gentamicin (47.1%), cefotaxime (43.15%), penicillin (30.8%), trimethoprim-sulphamethoxazole (25.5%), vancomycin (23.5%), and ampicillin (17.3) (Table 3). Multi-drug resistance (resistance two or more drugs) was observed in all isolated bacteria.

**Table 3:** Antibiotics Sensitivity Pattern of Bacterial isolates at Hawassa Referral and Teaching Hospital, 2012

**Bacterial isolates**	**No. of strains sensitive to Antibiotics (%)**							
	**No.**	**AMP**	**CN**	**P**	**SXT**	**NOR**	**VA**	**CTX**
***Staphylococcus auerus***	7	0(0.0)	2(28.6)	2(28.6)	4(57.1)	4(57.1)	6(85.7)	4(57.1)
**CoNS**	19	2(10.5)	8(42.1)	6(31.6)	4(21.1)	10(52.6)	2(10.5)	7(36.8)
***Escherichia coli***	16	5(31.2)	9(56.2)	-	3(18.8)	13(81.2)	3(18.8)	9(56.2)
***Citrobacter *** **species**	1	0(0.0)	1(100.0)	-	0(0.0)	0(0.0)	0(0.0)	0(0.0)
***Klebsiella *** **species**	3	1(33.3)	3(100.0)	-	1(33.3)	2(66.7)	1(33.3)	1(33.3)
***Pseudomonas aeruginosa***	1	0(0.0)	1(100.0)	-	0(0.0)	1(100.0)	0(0.0)	0(0.0)
***Enterobacter *** **species**	4	1(25.0)	0(0.0)	-	1(25.0)	3(75.0)	0(0.0)	1(25.0)
**Total**	51	9(17.6)	24(47.1)	8(30.8)	13(25.5)	33(64.7)	12(23.5)	22(43.1)

CoNS = coagulase negative Staphylococci; AMP = ampicillin; CN = gentamicin; P = penicillin; SXT = trimethoprim-sulphamethoxazole; NOR = norfloxacin; VA = vancomycin; CTX = cefotaxime.

## Discussion

Pregnant women are at increased risk of UTI but in many cases, infection remains asymptomatic [2]. In this study, the prevalence of asymptomatic urinary tract infection among pregnant women attending antenatal clinic of Hawassa Teaching and Referral Hospital was 18.8%, which is similar with previous work in northwest Ethiopia (Bahirdar) [16]. It is, however, higher than results from southwest (Jimma) [10], northwest (Gondar) [17], and central Ethiopia (Addis Ababa) [11], where rates ranging from 6 to 10.6% were reported. Our finding is lower than the figure reported from Iran (29.1%) [18] and Sagamu (23.9%) [19], Benin (45.3%) [20] and Abakaliki cities of Nigeria (78.7%) [21]. The varying prevalence of ASB from one country to another and among regions of the same country might be due to difference in risk factors with geographical areas.

The highest rate of ASB was reported in the age group ≥ 35 years and the lowest was in the age group 25–34 years. Similarly, higher infection rates were shown in the age group 25–29 years elsewhere [22,23]. Moreover, similar to previous findings by Okonko *et al.*[22] and Turpin *et al.*[23], the prevalence of asymptomatic bacteriuria has no significant association with trimester.

The predominant bacterial isolates observed in this study were CoNS (32.6%) followed by *E. coli* (26.1%), and *S. auerus* (13%). In agreement, Rahimkhani *et al*. [18] reported *S. epidermidis* (36%) and *E. coli* (20%) as prevailing bacterial isolates. The predominance of *E. coli* was also consistently reported by others [11,16-18,21,22,24]. High prevalence rate of *staphylococci* (45.6%) in this study were surprising result. This could be due to poor genital hygienic practices by pregnant women who may find it difficult to clean their anal or genital region properly after defecating or passing urine.

In this study, the traditional semi-quantitative bacterial counts (s-QBC) cut-off of value 10^5^ CFU/ml was used to define significant bacteriuria. However, recent studies have reported that this cut-off is insensitive and inappropriate for some clinical conditions and proposed lower s-QBC value cut-off, 10^3^ CFU/ml [25,26]. Tan *et al.* also showed that using a lower threshold of ≥10^3^ s-QBC would be appropriate for diagnosis of group B *streptococcus* (GBS) urinary tract infection [25]. The absence of GBS in our study, in contrast to others findings [5,27,28], may be due to the higher cut-off we used.

The susceptibility of bacterial isolates against norfloxacin, gentamicin and cefotaxime was 64.7%, 47.1% and 43.1% respectively. However, slightly higher susceptibility rates were reported for these antibiotics in Gondar [29] and Addis Ababa [11], which probably be due to widespread misuse of antibiotics in those areas.

## Conclusion

In conclusion, the prevalence of ASB in pregnant women was relatively higher than previous findings in Ethiopia. This study also showed, most of bacterial isolates are resistant against commonly used antimicrobial agents. Therefore, routine laboratory diagnosis of ASB in pregnant women and providing appropriate treatment would be needed to reduce its complications.

## Abbreviations

ABU: Asymptomatic bacteruria; UTI: Urinary tract infection; CFU: Colony forming unit; CoNS: Coagulase negative Staphylococci; GBS: Group B streptococcus; s-QBC: Semi-quantitative bacteruria count.

## Competing interests

We declare that we have no competing interests.

## Authors’ contributions

ET was the principal investigator for the study: TS, MT, BK and YM contributed to the design of the study. ET and YM carried out laboratory work. TS and ET performed statistical analysis and interpreted the result. All authors contributed to the write up of the manuscript.
